# Characterization of a Unique Novel LORF3 Protein of Duck Plague Virus and Its Potential Pathogenesis

**DOI:** 10.1128/jvi.01577-22

**Published:** 2023-01-04

**Authors:** Bingjie Shen, Peilin Ruan, Anchun Cheng, Mingshu Wang, Wei Zhang, Ying Wu, Qiao Yang, Bin Tian, Xumin Ou, Sai Mao, Di Sun, Shaqiu Zhang, Dekang Zhu, Renyong Jia, Shun Chen, Mafeng Liu, Xin-Xin Zhao, Juan Huang, Qun Gao, Yanling Yu, Ling Zhang, Leichang Pan

**Affiliations:** a Institute of Preventive Veterinary Medicine, Sichuan Agricultural University, Chengdu City, Sichuan, China; b Sinopharm Yangzhou VAC Biological Engineering Co., Ltd., Yangzhou City, China; c Key Laboratory of Animal Disease and Human Health of Sichuan Province, Sichuan Agricultural University, Chengdu City, Sichuan, China; d Avian Disease Research Center, College of Veterinary Medicine, Sichuan Agricultural University, Chengdu City, Sichuan, China; Lerner Research Institute, Cleveland Clinic

**Keywords:** duck plague virus, LORF3, unique genes, growth kinetics, virus replication, pathogenesis

## Abstract

Duck plague virus (DPV) is a high-morbidity fowl alphaherpesvirus that causes septicemic lesions in various organs. Most DPV genes are conserved among herpesviruses, while a few are specific to fowl herpesviruses, including the *LORF3* gene, for which there is currently no literature describing its biological properties and functions. This study first addressed whether the LORF3 protein is expressed by making specific polyclonal antibodies. We could demonstrate that DPV *LORF3* is an early gene and encodes a protein involved in virion assembly, mainly localized in the nucleus of DPV-infected DEF cells. To investigate the role of this novel LORF3 protein in DPV pathogenesis, we generated a recombinant virus that lacks expression of the LORF3 protein. Our data revealed that the LORF3 protein is not essential for viral replication but contributes to DPV replication *in vitro* and *in vivo* and promotes duck plague disease morbidity and mortality. Interestingly, deletion of the LORF3 protein abolished thymus atrophy in DPV-vaccinated ducks. In conclusion, this study revealed the expression of avian herpesviruses-specific genes and unraveled the role of the early protein LORF3 in the pathogenesis of DPV.

**IMPORTANCE** DPV is a highly lethal alphaherpesvirus that causes duck plague in birds of the order Anseriformes. The virus has caused huge economic losses to the poultry industry due to high morbidity and mortality and the cost of vaccination. DPV encodes 78 open reading frames (ORFs), and these genes are involved in various processes of the viral life cycle. Functional characterization of DPV genes is important for understanding the complex viral life cycle and DPV pathogenesis. Here, we identified a novel protein encoded by *LORF3*, and our data suggest that the LORF3 protein is involved in the occurrence and development of duck plague.

## INTRODUCTION

Duck plague (DP), also known as duck viral enteritis, is an acute, febrile, and septic infectious disease that can infect ducks, geese, and a variety of Anseriformes. It is caused by the duck plague virus0 (DPV)/anatid herpesvirus-1 (AnHV-1) of the genus *Mardivirus*, family Herpesviridae, and subfamily Alphaherpesvirinae (1, 2). DPV infects waterfowl of all ages, causes extremely high mortality, and is distributed worldwide, with migratory waterfowl playing a critical role in its intra- and intercontinental spread (2). Mature DPV has a typical herpesvirus particle structure, which is spherical, has a diameter of ~150 to 300 nm, and consists of a capsule, cortex, capsid, and core from outside to inside (3, 4). The full-length genome of the Chinese virulent CHv strain of duck plague virus is 162,175 bp and contains 78 open reading frames (ORFs). The genome structure is mainly composed of 5′ to 3′ orientation as UL-IRS-US-TRS. Among them, 65 ORF boxes are in the UL region, 11 ORF boxes are in the US region, and 2 ORF boxes are in the IRS and TRS regions (5).

Among the herpesviruses, there is a type of virus that only infects birds but not mammals, called avian herpesvirus, which includes Marek's disease virus (MDV), DPV, infectious laryngotracheitis virus (ILTV), turkey herpesvirus (HVT), pigeon herpesvirus (PiHV), psittacid herpesvirus (PsHV), varanid herpesvirus (VHV), podargid alphaherpesvirus 1 (PodHV1), and cacatuid alphaherpesvirus 1 (CacHV1) (6). Avian herpesvirus genes are divided into two categories: one is homologous to other herpesviruses, and these genes account for the majority of the genome; the other is the unique genes of avian herpesviruses, named *LORF*, *RLORF*, *SORF*, and *RSORF* according to the location of the initiation codon in the genome, which is related to the unique biological characteristics of the viruses (7, 8). It is reported that there are 5 unique genes in avian herpesviruses whose genome has been sequenced so far, 4 of which are located in the UL region, and 1 is located in the US region, which are named *LORF2* (*vLIP*), *LORF3*, *LORF4* (*LORF9*), *LORF5* (*LORF11*), and *SORF3* according to the MDV-homologous genes (9). In addition, there is a class of special genes that only exists in the inverted repeat region of a specific virus, such as the *Meq* gene of MDV-1 and the *BclII* gene of MeHV-1, which usually play a crucial role in the pathogenicity of the virus (10).

Studies have shown that the unique genes *LORF2*, *LORF4*, *LORF5*, *RLORF2*, *RLORF4*, and *RLORF7* are all related to virus virulence. Knockout of the MDV *LORF2* gene reduces the incidence and lethality of visceral lymphoma in susceptible chickens (11). The MDV LORF4 protein can interact with the β chain and γ chain of major histocompatibility complex (MHC) class II proteins to escape the host immune response, and deletion of LORF4 can reduce the virulence of MDV (12, 13). The *LORF5* gene-deleted MDV significantly reduces the lethality of chickens and does not induce tumor lesions in chickens. In the duck plague virus, the *LORF5* gene is closely related to the cell-to-cell transmission and replication of the virus (14, 15). The *RLORF2* gene encodes a viral CXC chemokine called vIL-8, and studies have shown that MDV vIL-8 deletion mutants are significantly less virulent and provide effective protection against highly virulent strains in susceptible chickens (16, 17). The protein encoded by *RLORF4* can inhibit cGAS-STING-mediated activation of NF-κB, thereby inhibiting the production of interferon beta (IFN-β), so MDV can use this function of the *RLORF4* gene to achieve immune evasion from the host (18, 19). The Meq protein encoded by the *RLORF7* gene is considered the main oncoprotein of MDV and can induce tumor transformation of T cells through various mechanisms such as inducing host cell apoptosis (20). Many live attenuated vaccines using MDV as a vector have been developed by using *RLORF7* gene deletion strains.

In this study, we demonstrate that the DPV *LORF3* gene is an early gene that encodes a novel protein as a viral structural protein, which is reported for the first time. The viral LORF3 protein is located in the nucleus of DEF cells and does not change over time. We constructed a *LORF3* gene deletion virus (DPV CHv-ΔLORF3) and its revertant (DPV CHv-ΔLORF3 Rev) using the scarless Red recombination system. The data show that the *LORF3* gene is dispensable for DPV invasion, replication, assembly, and release formation *in vitro* but contributes to DPV cell-to-cell spread and replication. In addition, pLORF3 contributes to the occurrence and development of duck plague and the pathological atrophy of the thymus in infected ducks.

## RESULTS

### Duck plague virus LORF3 gene encodes a novel protein.

**FIG 1:**
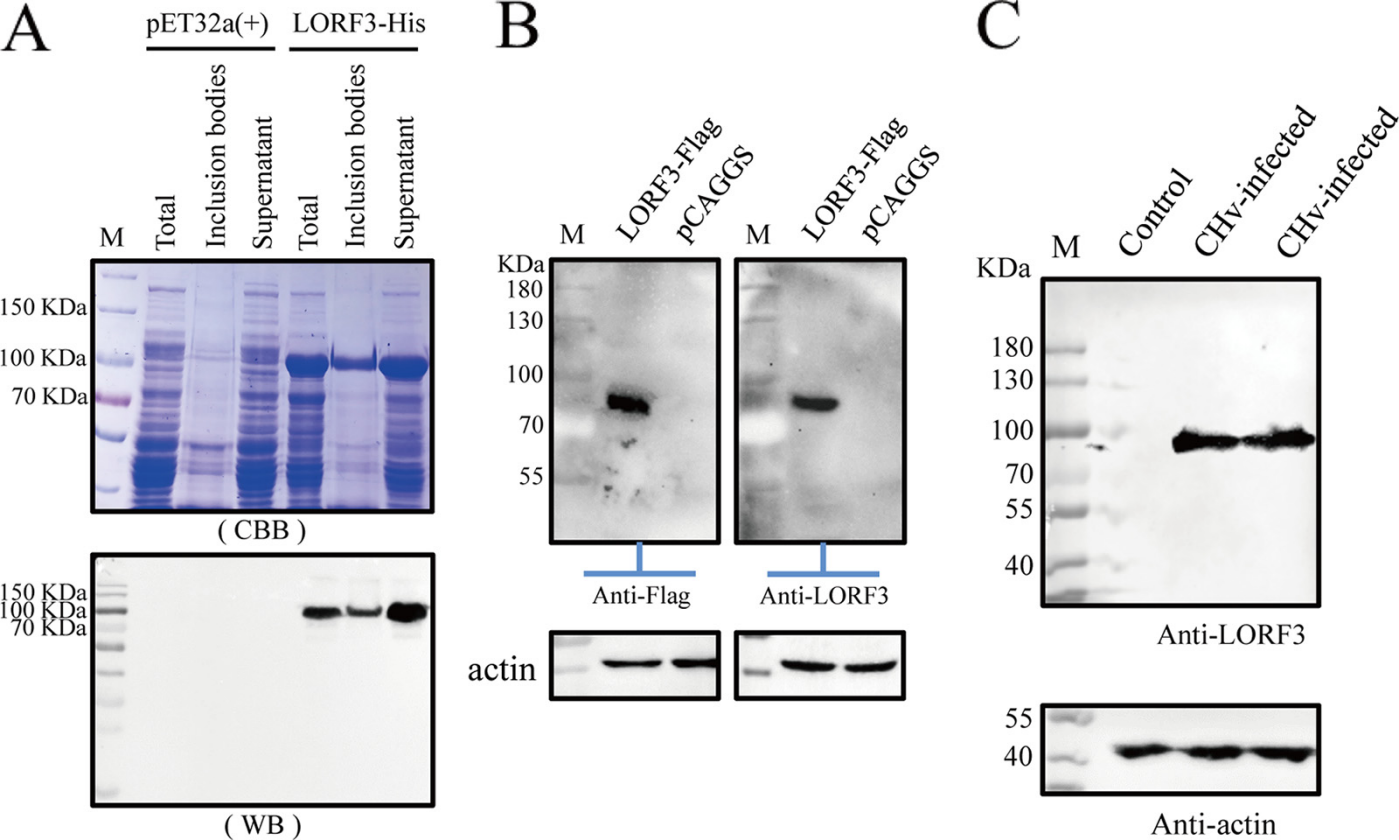
LORF3 protein polyclonal antibody preparation and expression analysis of DPV LORF3. (A) Expression and identification of LORF3-His fusion protein exist in soluble and insoluble forms. (B) Rabbit anti-LORF3 polyclonal antibody recognizes the expression of the LORF3 eukaryotic protein by Western blotting. DEF cells were transfected with PCAGGS-LORF3-Flag and harvested at 36 hpi. Protein was specifically recognized using a rabbit anti-LORF3 antibody (1:1,000), and the Flag-tagged antibody (1:3,000) on the left was used as a control. (C) Detection of protein expression of the *LORF3* gene in virus-infected cells by Western blotting. DEF cells were infected with indicated viruses and harvested at 24 hpi. Proteins were detected using a rabbit anti-*LORF3* protein polyclonal antibody.

The expressed product was a LORF3-His fusion protein, which exists in soluble and insoluble forms and was not detected in the carrier control culture (Fig. 1A). Next, the fusion protein was purified by SDS-PAGE gel cutting and then administered to rabbits with Freund’s adjuvant (catalog no. BY-s2; Sigma, USA) to prepare specific anti-LORF3 polyclonal antibodies. pLORF3 in eukaryotic plasmid pCAGGS-LORF3-Flag-transfected cells reacted strongly with this specific polyclonal antibody (Fig. 1B, right), which was the same as the result of the Flag-tagged antibody blot (Fig. 1B, left). To further validate the expression of the unique gene *LORF3*, we performed Western blotting (WB) by rabbit anti-LORF3 polyclonal antibody and could confirm the expression of DPV *LORF3* (Fig. 1C). Taken together, we concluded that a novel protein-encoding gene named *LORF3* was first confirmed.

### The *LORF3* gene may be an early gene encoding a structural protein.

**FIG 2:**
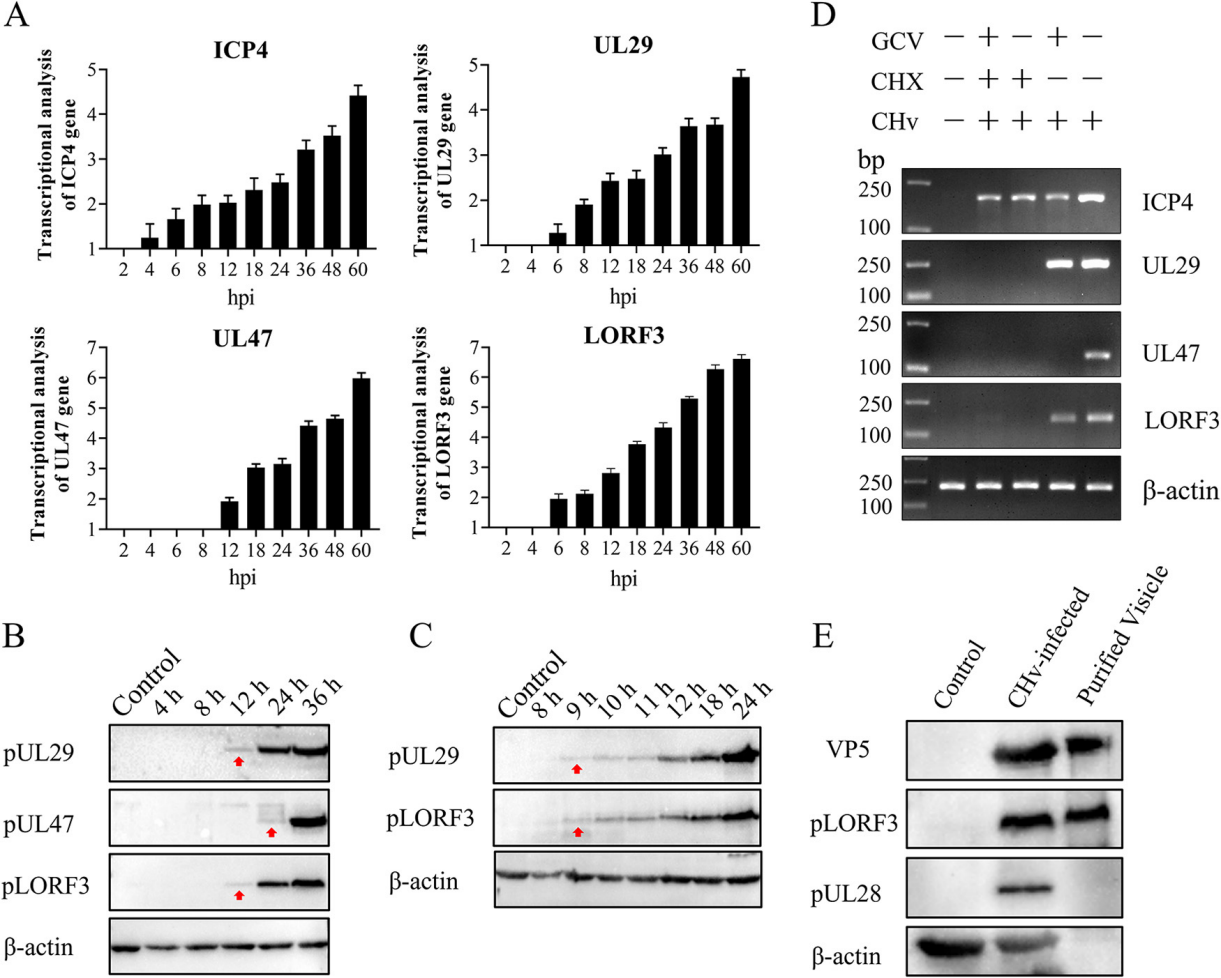
Analysis of *LORF3* genotype and structural properties of the encoded protein. (A) Total RNA isolated from mock cells and DPV-infected cells at different times was analyzed using RT-qPCR and normalized to β-actin, and the fold change of the mock was plotted. *ICP4*, *UL29*, and *UL47* were used as IE, E, and L gene controls, respectively. (B) Expression analysis of the *LORF3* gene product in DPV-infected DEFs at different time points. Samples and gels from the same experiment were processed in parallel. (C) LORF3 protein expression was analyzed for more detail and time points. Samples and gels from the same experiment were processed in parallel. (D) Cells treated with GCV and CHX were infected with DPV for 18 hpi, and the isolated RNA was reverse transcribed into cDNA as the template for PCR-nucleic acid electrophoresis analysis. The properties of the LORF3 gene were identified by the expression of each type of gene. (E) Purified DPV virions were identified using rabbit anti-LORF3 antibody sera. The LORF3 protein is present in the components of virions, and no bands were present in the DEF cell group. The viral structural protein VP5 and the nonstructural protein UL28 were set for comparison.

To verify the expression stage of the *LORF3* gene, we investigated the transcript level of *LORF3* at different time points, 2, 4, 6, 8, 12, 18, 24, 36, 48, and 60 h postinfection (hpi) and in mock-infected DEF cells by reverse transcription-quantitative PCR (qRT-PCR). The immediate early (IE) gene *ICP4*, early (E) gene *UL29*, late (L) gene *UL47*, and β-actin were also detected as controls. *LORF3* transcription was first detected at 6 hpi and increased over time, the same as the transcription of the early gene *UL29* (Fig. 2A). Meanwhile, the mRNA of the immediate early gene *ICP4* could be detected at 4 hpi, while the late gene *UL47* was only detected at 12 hpi (Fig. 2A). We also investigated the expression of pLORF3 at different time points (4, 8, 12, 24, and 36 hpi) in infected and mock-infected DEF cells. We found that pLORF3 was first detected at 12 hpi and expressed simultaneously with the early gene (Fig. 2B). To further illustrate the problem, we tested whether the LORF3 protein was expressed simultaneously with the UL29 protein by narrowing the time interval (8, 9, 10, 11, 12, 18, and 24 hpi) and obtained similar results (Fig. 2C). Drug inhibition experiments were performed to determine *LORF3* gene types, specifically, total RNA isolated from DEFs with coculture of ganciclovir-cycloheximide (GCV-CHX) and DPV for PCR analysis. As shown in Fig. 2D, DPV *LORF3* was detected after treatment with the nucleic acid synthesis inhibitor GCV but not after treatment with the protein synthesis inhibitor CHX, which was the same as the measured early gene *UL29*. We concluded from the above-described results that the DPV *LORF3* is an early gene.

To elucidate the structural properties of *LORF3*-encoded amino acids, we purified virions by ultracentrifugation and detected them with specific antibodies by WB. Furthermore, the DPV pLORF3 band was detected in virus-infected cells and virion particles (Fig. 2E). The reported structural component VP5, nonstructural component UL28, and β-actin were also detected as controls. Furthermore, mass spectrometry was used to identify the protein content of extracellular virions. The results showed that pLORF3 was present in mature extracellular virions (Table 1), and LORF3 was likely to be involved in the particle composition of DPV.

**TABLE 1:** Viral content of DPV extracellular virions (partial)

Protein	Description	Score	Mass	Matches	Sequence	emPAI^*a*^	GenPept accession no.
UL44 (27)	Glycoprotein C	97	47,836	6 (3)	6 (3)	0.22	AJG04885
LORF3	LORF3 (anatid alphaherpesvirus 1)	67	54,300	6 (3)	3 (1)	0.19	AJG04932

a emPAI, exponentially modified protein abundance index.

### Localization of pLORF3 in DEF cells.

**FIG 3:**
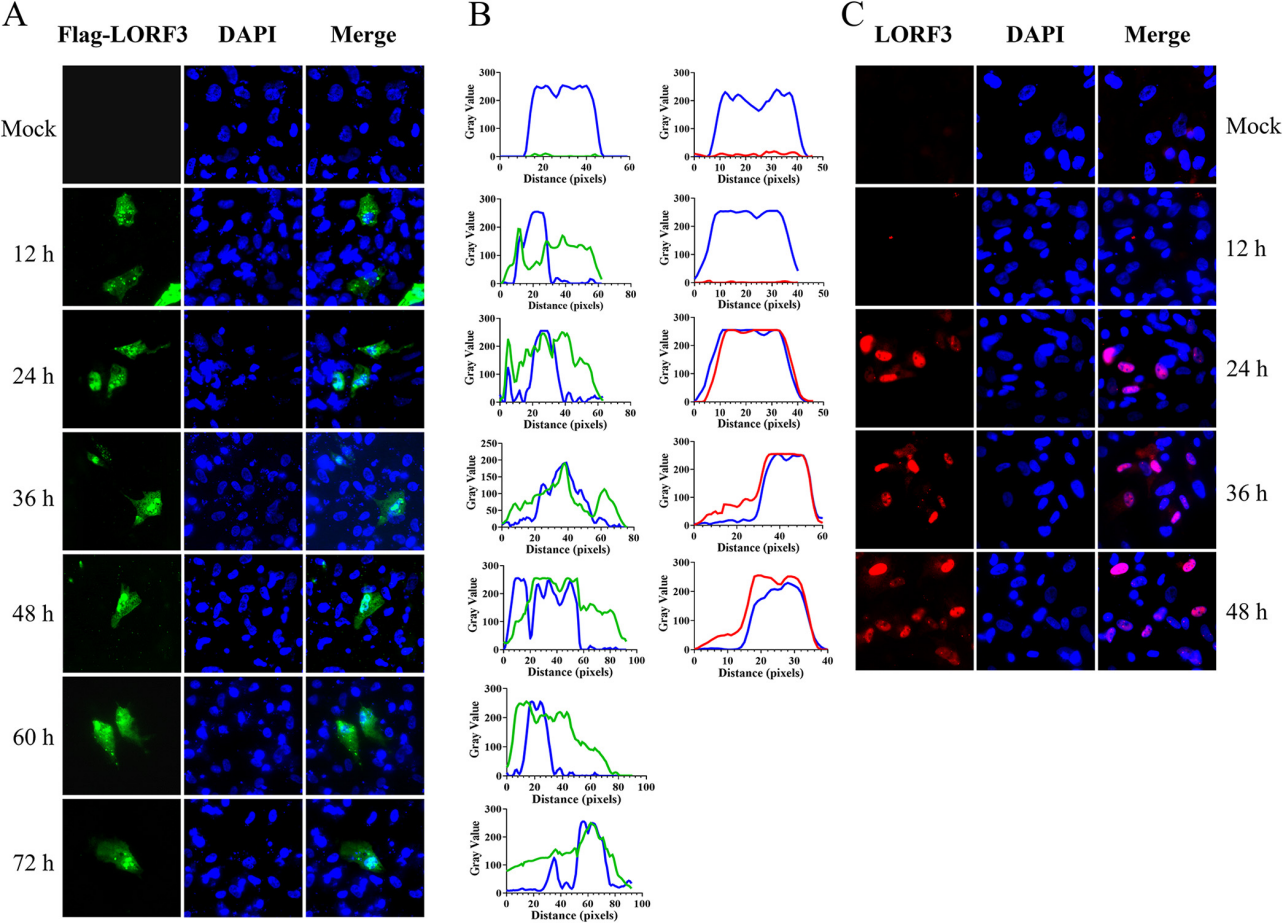
Intracellular localization and distribution of LORF3-encoded protein. (A) Localization of overexpressed pLORF3 (green in images) in the mock group includes DEF cells transfected with pCAGGS empty, using Flag-tagged monoclonal antibody (MAb) and Alexa Fluor 488 goat anti-mouse IgG. DAPI, 4′,6-diamidino-2-phenylindole. (B) Fluorescence waveform analysis diagram of protein expression. Analysis was performed using ImageJ and GraphPad Prism8 software. (C) Representative DPV pLORF3 localization (red in images). The mock group included untreated DEF cells using rabbit anti-LORF3 polyclonal antibody and Alexa Fluor 594 goat anti-rabbit IgG (scale bar, 10 μm).

To analyze the intracellular localization of the pLORF3, immunofluorescence assay (IFA) was performed on DEF cells transfected with pCAGGS-LORF3-Flag or infected with DPV. The results showed that Flag-pLORF3-specific fluorescence (green) was localized in the whole cells and punctually distributed in the nucleus, and the signal became progressively stronger over time (Fig. 3A). The protein accumulated in the nucleoli and appeared diffuse (~12 to 72 hpi). No fluorescence was observed in cells transfected with pCAGGS empty vector plasmid. In contrast, however, the pLORF3-specific fluorescence (red) was primarily localized in the nucleus in DPV-infected cells (Fig. 3C). As described above, we found that the subcellular localization of pLORF3 was altered in plasmid-transfected and virus-infected cells. This changing trend could also be seen in the waveform of the fluorescence value (Fig. 3B).

### Construction of recombinant duck plague virus.

**FIG 4:**
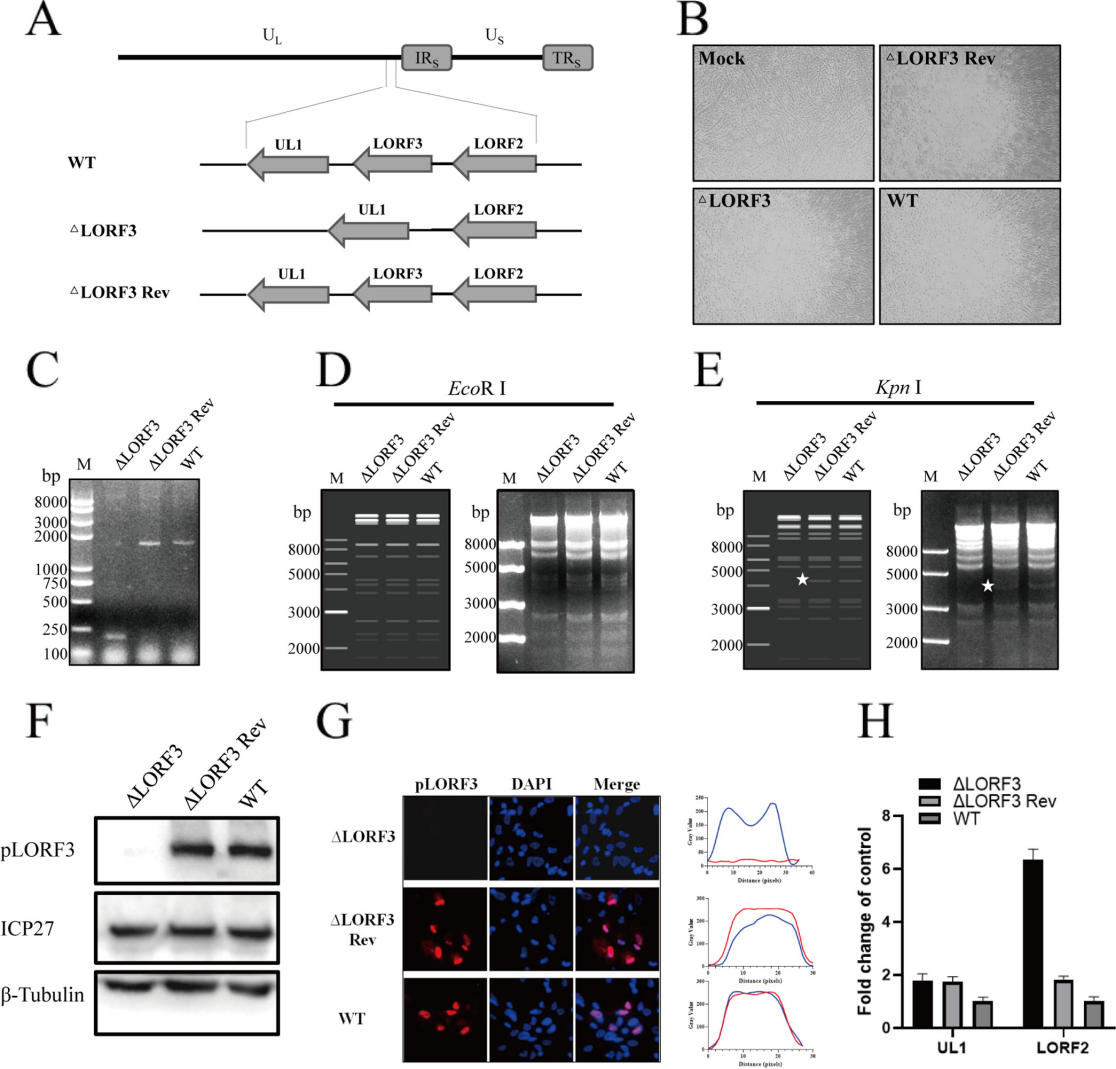
Construction and identification of recombinant viruses. (A) Focus on the LORF3 region is shown with the positive surrounding genes for LORF2. The LORF3 full-length fragment was deleted in the overall frame. (B) BAC-free LORF3 deletion mutants (ΔLORF3), reversion mutants (ΔLORF3 Rev), and parental strains (WT) were harvested after rescue and purification. (C) Identification of recombinant viruses by PCR analysis. (D) RFLP analysis. Infectious cloned plasmids were treated with QuickCut EcoRI and imaged by 1% gel electrophoresis, and no visible differences were found. (E) RFLP analysis. Infectious cloned plasmids were treated with QuickCut KpnI and imaged by 1% gel electrophoresis, and the deletion strain lacks a band of about 4 kb (the place indicated by the asterisk). (F) Western blot analysis of LORF3 protein expression. Recombinant virus-infected cell lysates were subjected to Western blotting, and protein expression was detected using an anti-LORF3 polyclonal antibody. β-Tubulin was used as a control. (G) Detection of pLORF3 in virus-infected cells by IFA. DEF infected with indicated viruses were fixed at 24 hpi and then stained with a rabbit anti-LORF3 polyclonal antibody (red), and nuclei were visualized using DAPI (blue) (scale bar, 10 μm). (H) Effects of LORF3 deletion on peripheral gene expression.

To determine the role of DPV pLORF3 in virus replication, we generated a recombinant virus in which the full-length gene fragment was knocked out without scarring (DPV CHv-ΔLORF3). To confirm the abrogation of protein expression, we also generated a LORF3 gene recovery strain (DPV CHv-ΔLORF3 Rev), subsequently (Fig. 4A and B). Positive bacterial artificial chromosome (BAC) clones were confirmed by PCR amplification and restriction fragment length polymorphism (RFLP) analysis. The PCR amplification product of the LORF3-deleted strain was 200 bp, while the revertant and parental strain were 1,643 bp (Fig. 4C). Enzyme cleavage maps displayed that plasmids had no difference after digestion by QuickCut EcoRI (Fig. 4D). Simultaneously, CHv-ΔLORF3 lacked a nucleic acid band of about 40,000 bp after digestion by QuickCut KpnI compared to CHv-ΔLORF3 Rev and wild type (WT) (Fig. 4E), which is the same as the predicted result. To verify that LORF3 knockout indeed abrogated LORF3 expression, we analyzed CHv-ΔLORF3-infected DEF by Western blotting and IFA (Fig. 4F and G). Production of the LORF3 protein (pLORF3) was completely abolished in CHv-ΔLORF3-infected cells. In these experiments, the DPV ICP27 protein was used as a viral control, and the cellular β-tubulin was used as a loading control. In conclusion, the recombinant viruses were correctly constructed. On the contrary, we were surprised to find that *LORF3* deletion did not affect the expression of its downstream gene *UL1* but, in turn, upregulated the transcription of its upstream gene LORF2, which has been reported to be a virulence-related gene (Fig. 4H).

### Growth kinetics of LORF3 mutants *in vitro*.

**FIG 5:**
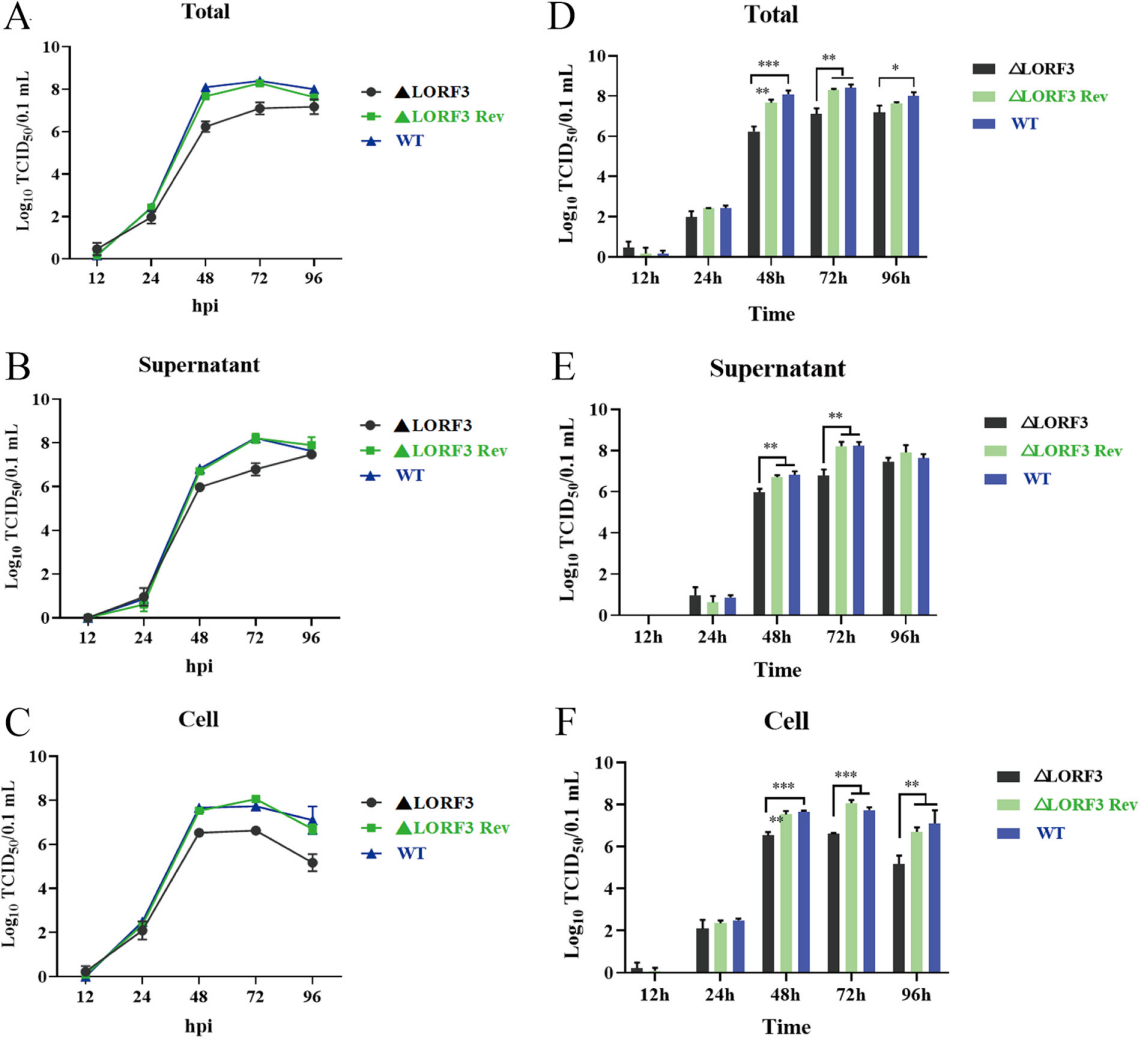
Multistep growth kinetics assays of indicated recombinant viruses. (A) Virus replication level curve in total samples. (B) Virus replication level curve in the supernatant medium. (C) Virus replication level curve in cell samples. (D to F) Statistical analysis of the difference of virus titer in the total (D), supernatant (E), and cell (F) at each time point. Asterisks indicate significant differences from the WT virus. Data are shown as mean virus titer of three independent experiments each with standard deviations (***, *P < *0.001; **, *P < *0.01; *, *P < *0.05).

Next, we investigated if pLORF3 plays a role in DPV replication through multistep growth kinetics assays. Growth kinetics revealed that the abrogation of pLORF3 did not kill virus replication but attenuated the replication efficiency of the virus *in vitro* compared to the wild-type (WT, DPV, CHv) and reversion mutant virus (Fig. 5). At ~12 to 96 h after infection, ΔLORF3, ΔLORF3 Rev, and CHv viruses had the same replication pattern, and they all showed an upward trend with time; the virus titer reached its peak at 72 h, then, the virus replication gradually slowed down and reached a plateau, and even the virus content in the cells (Fig. 5C and F) began to decrease. Next, we analyzed the difference in the data using GraphPad Prism version 8 (San Diego, CA, USA) and found that mature virions were significantly reduced in supernatants (Fig. 5B and E), cells (Fig. 5C and F), and whole samples (Fig. 5A and D) after infection with the ΔLORF3 deletion mutant compared to CHv, up to about 27-, 13-, and 20-fold, respectively. The growth curve of the ΔLORF3 Rev-restored virus was always consistent with the CHv parental strain. We conclude from the results that this novel protein encoded by LORF3 is not essential for viral replication but promotes the replication of the duck plague virus *in vitro*.

### pLORF3 affects the replication of duck plague virus *in vitro*.

**FIG 6:**
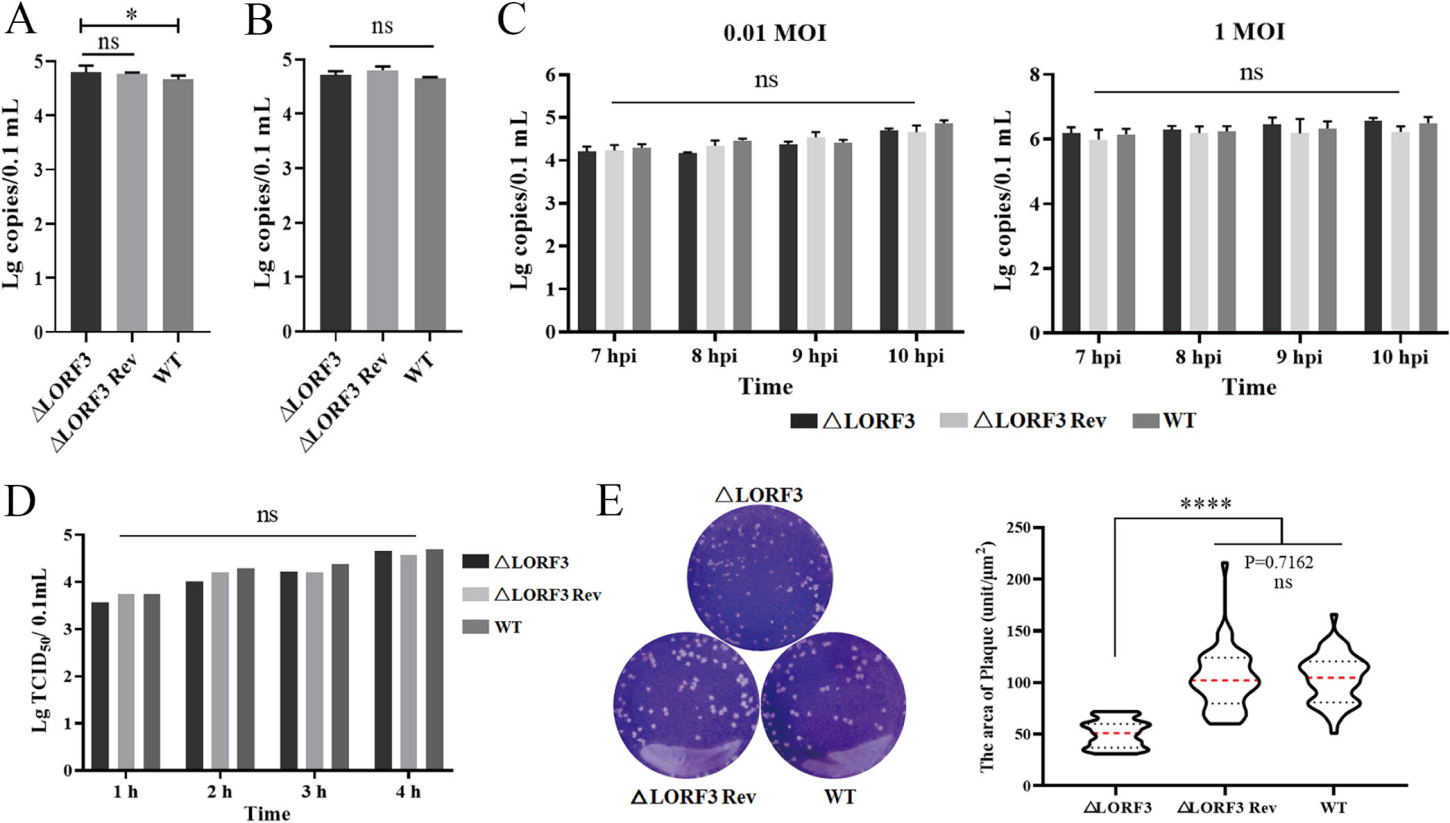
LORF3 in the viral adsorption, invasion, replication, and release processes and cell-to-cell spread. (A) DEF cells were infected with the indicated viruses at 4°C, and cell samples were collected after 2 hpi to detect the copies and analyze the adsorption efficiency of viruses. (B) After viral adsorption was completed, the cells were incubated at 37°C for 1 hpi, and the viruses in the cell were quantified to analyze the viral invasion efficiency. (C) qPCR assays for viral genome copies in cells infected with low- and high-dose DPV in consecutive periods. (D) Cells were replaced with fresh maintenance solution after DPV infection, the supernatant was collected every 1 hpi, and the release of mature virions was detected by virus titer. (E) Crystal violet assay to test the cell-to-cell spread. Representative plaques showing 50 plaques per sample were measured to quantify the results at the right. Plates were scanned, and plaque diameters were measured in Image J (ns, *P > *0.05; *, *P < *0.05; ****, *P < *0.0001).

The life cycle of the herpesvirus is roughly divided into adsorption, invasion, replication, release, assembly, and transmission. The viral adsorption and invasion experiments were conducted to explore whether pLORF3 is involved in the virus penetration process and investigate the function of pLORF3 in the viral life cycle. The viral genome copies between the mutant viruses and the WT group were analyzed by qPCR test. As shown in Fig. 6A, there were no significant differences in viral copy numbers between the ΔLORF3, ΔLORF3 Rev, and WT groups. Therefore, pLORF3 is not required for the adsorption process. Then, the virus invasion process was also detected (Fig. 6B), and the result was the same as the adsorption experiment. The above-described preliminary results indicate that pLORF3 is not involved in the early virus infection process. As an uncharacterized protein of avian herpesviruses, whether pLORF3 affects viral replication remains to be explored. By infecting with low and high doses of mutant viruses, we found that LORF3-deleted virus did not affect viral genome replication at all infectious doses (Fig. 6C). The virus release efficiency between the mutant viruses and WT was examined to investigate whether pLORF3 is involved in DPV release. The results showed that although pLORF3 was knocked out, the virus release efficiency was also the same as that of the revertant and WT (Fig. 6D). There were no significant differences in the titers of virus released to the outside the cells during the same period between the three groups. Therefore, it is preliminarily judged that LORF3 does not affect the virus release stage of the virus life cycle.

Subsequently, we verified viral plaque morphometry and found that compared with WT-infected cells, the plaque area was significantly reduced after ΔLORF3-infected cells, but there was no significant difference in ΔLORF3 Rev-infected cells (Fig. 6E). Then, 50 plaques were randomly selected from each group, the area size was calculated by ImageJ software, and statistical analysis was performed by GraphPad Prism 8.0.2 software. As shown in Fig. 6E, compared with the WT, the plaque area formed by the ΔLORF3 was reduced, about 76.7% of that of WT, while the plaque area formed by the ΔLORF3 Rev was not significantly different from that of WT. Therefore, we preliminarily judged that pLORF3 may be involved in the late stage of DPV replication.

### pLORF3 promotes the pathogenesis and lethality of DP.

**FIG 7:**
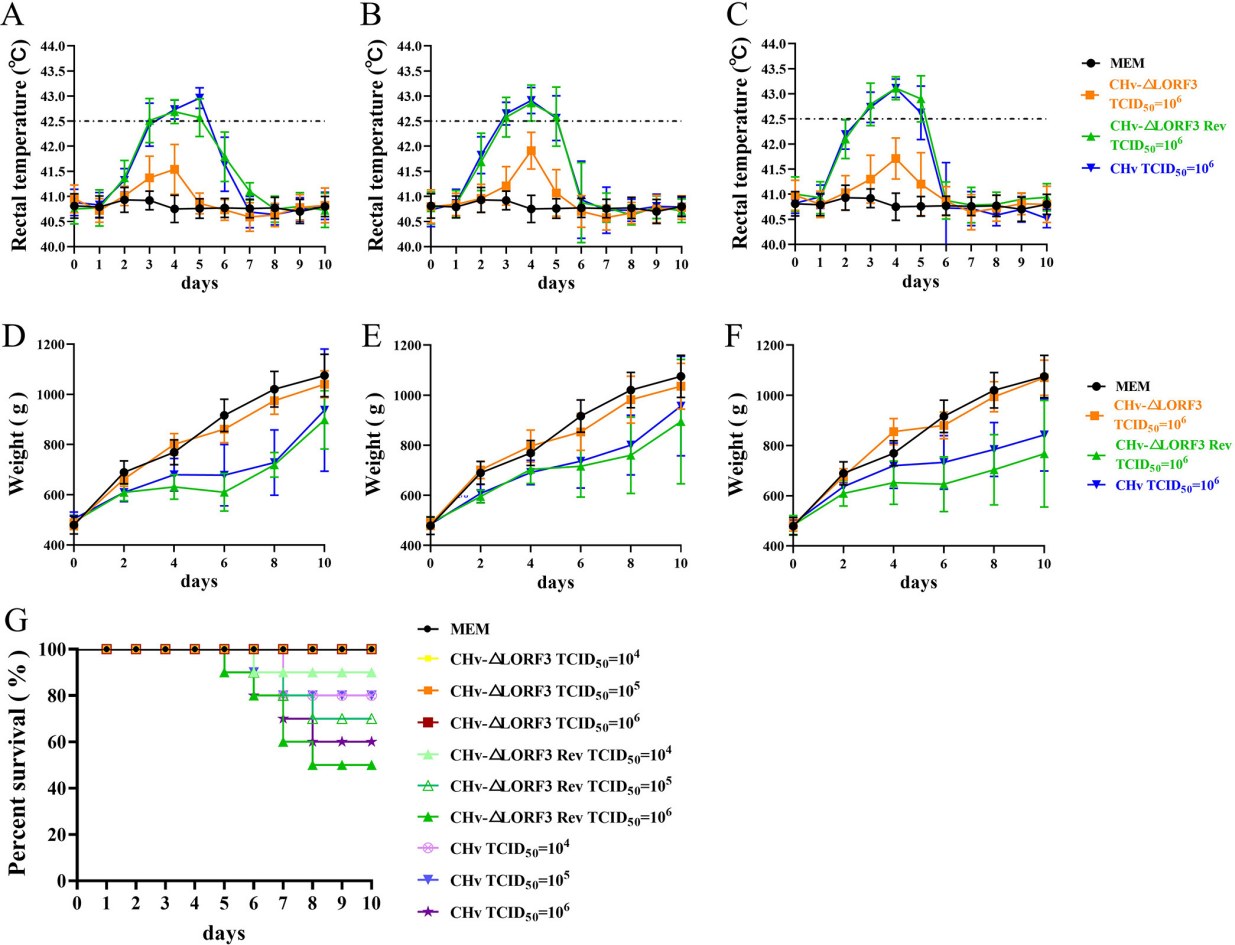
Pathogenesis of deletion, reversion, and parental viruses. (A to C) Rectal temperature of DPV-inoculated ducks. The temperature of all ducks inoculated with 10^4^, 10^5^, and 10^6^ TCID_50_ doses were monitored daily for 10 dpi, and the trend curve for each group was plotted. (D to F) Body weight gain rate of ducks inoculated with DPV. The weights of all ducks infected with 10^4^, 10^5^, and 10^6^ TCID_50_ doses were monitored for 10 days, and weight trend curves were drawn. (G) Survival curve of DPV inoculated ducks. Mortalities of all groups were recorded daily for 10 dpi, and the survival rates for each group were graphed.

To elucidate if pLORF3 is involved in DPV pathogenesis, we infected 14-day-old ducklings, which were tested free of DPV and DPV antibodies, with 10^4^, 10^5^, and 10^6^ 50% tissue culture infectious dose (TCID_50_) of CHv-ΔLORF3, CHv-ΔLORF3 Rev, or CHv (WT) viruses and monitored them for 10 days. We found that the rectal temperature of all ducks in the ΔLORF3 group fluctuated within the normal range during the 10 days (Fig. 7A to C), and the body weight continued to increase rapidly, which was not significantly different from the control group (minimal essential medium [MEM] group) (Fig. 7D to F). Whereas the rectal temperature of ducks in the ΔLORF3 Rev and WT groups increased within ~2 to 6 days after the challenge (Fig. 7A to C), the body weight increased slowly or even negatively (Fig. 7D to F). The temperature in ducklings was positively correlated with inoculated virus titers, with higher inoculated virus titers associated with higher rectal temperatures in affected ducks (Fig. 7A to C). Mortality occurred in both WT and ΔLORF3 Rev-infected ducks at the lowest dose (10^4^ TCID_50_), while all ducks in the highest challenge dose (10^6^ TCID_50_) of ΔLORF3 and control groups survived within 10 days (Fig. 7G; Table 2). Thus, the minimum dose that caused animal death was more than 100-fold lower for the deletion strain than for the restorer and parental strains.

**TABLE 2:** Mortality statistics of ducks

Virus	Mortality (%) at TCID_50_ of:		
	10^4^	10^5^	10^6^
MEM (control)	0	0	0
DPV CHv-ΔLORF3	0	0	0
DPV CHv-ΔLORF3 Rev	10	30	50
DPV CHv (WT)	20	20	40

### Elimination of pLORF3 inhibits DPV-induced pantropic tissue damage.

**FIG 8:**
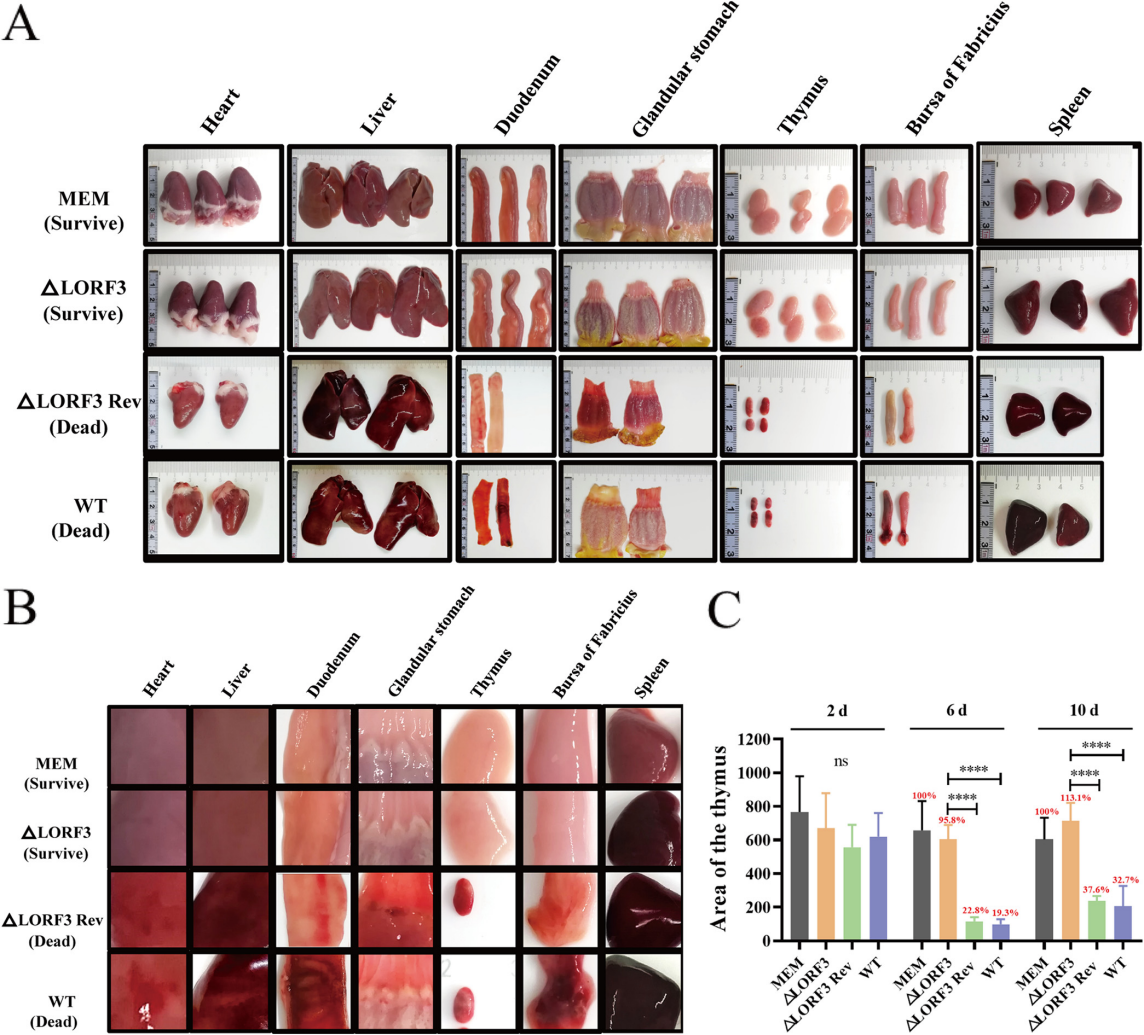
Autopsy histopathology of designated viruses-infected ducks. (A) Histopathological damage records of the dead ducks infected with revertant and parental strains and the surviving ducks infected with the deletion strain at 6 dpi. (B) Magnified view of the local lesion of the organs in panel A. (C) Thymus atrophy due to DPV infection. Each group's thymus area of ducks was scanned and measured with ImageJ within 10 days of the attack. Results are shown based on the thymus area of ducks in the control group as 100% (****, *P < *0.0001).

Next, to address whether pLORF3 contributes to DPV pathogenesis, we monitored the infected animals for tissue lesions and viral load throughout the experiment. Fourteen-day-old ducklings were infected with the highest dose (10^6^ TCID_50_) of ΔLORF3 deletion virus that did not cause death, and ΔLORF3 Rev and WT were set as controls. The death occurred in ΔLORF3 Rev- and WT-infected ducks. Necropsy observation revealed that the dead duck showed symptoms of systemic sepsis, including bleeding spots on the surface of the heart; severe congestion and necrosis of the liver; spleen enlargement, hemorrhage, and soft texture; thymic hemorrhage and atrophy; and duodenal and bursa hemorrhage (Fig. 8A and B). However, there were no deaths in the ΔLORF3 group. The necropsy of the surviving ducks revealed hemorrhage and enlargement of the spleen, slight hemorrhage in the thymus, and no obvious lesions in other organs (Fig. 8A and B). Interestingly, at the late stage of virus infection, the thymus in ΔLORF3 Rev- and WT-infected ducks shrank to only ~19.3% to 36.7% of that in the MEM group (Fig. 8C). The above-described results indicate that the LORF3 protein is involved in the pathogenesis of DPV, and the elimination of LORF3 protein inhibits DPV-induced thymus atrophy.

### Elimination of LORF3 impairs the replication capacity of DPV *in vivo*.

**FIG 9:**
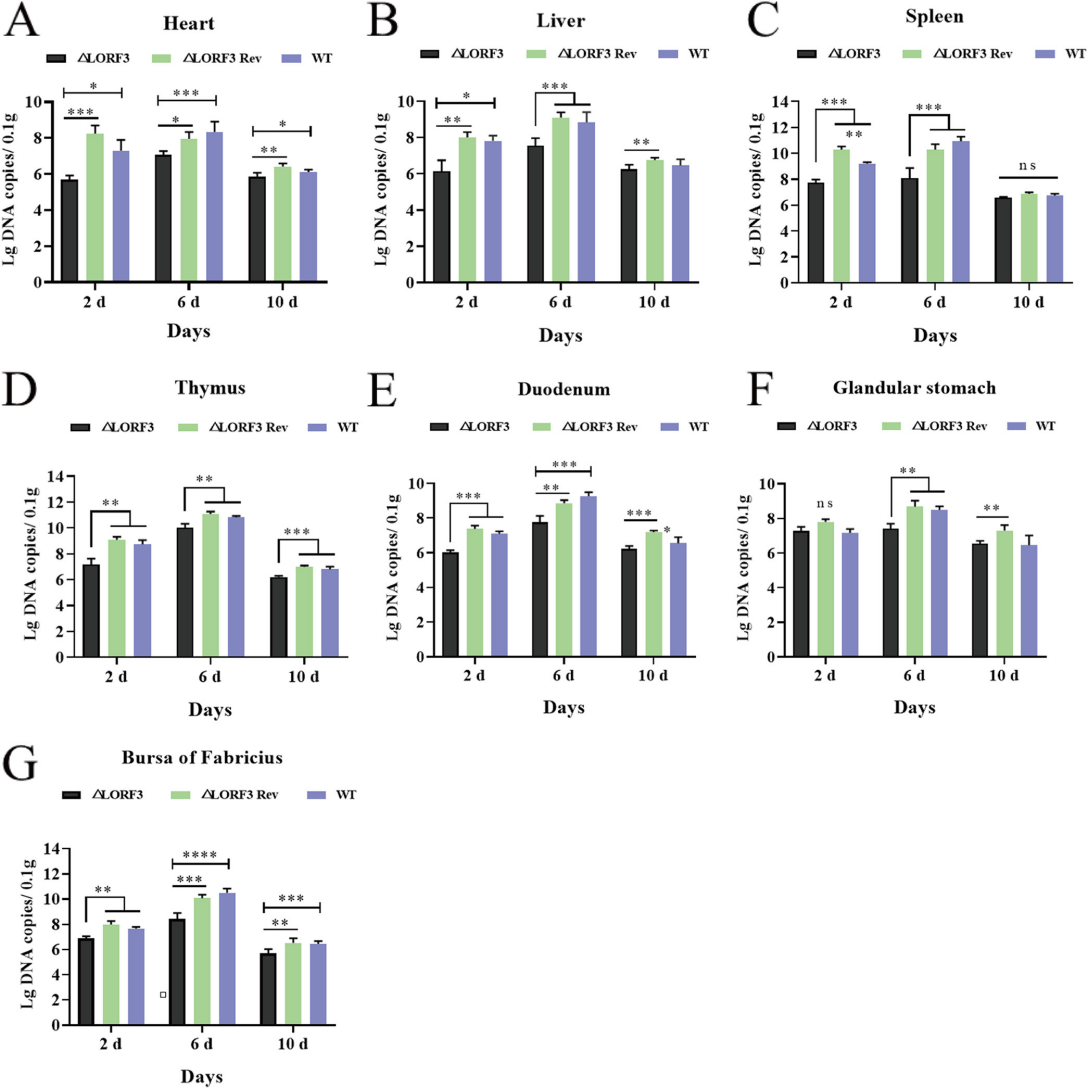
Replication characteristics of LORF3 mutants *in vivo*. (A to G) DPV genome copies of indicated viruses were quantified in the heart (A), liver (B), spleen (C), thymus (D), duodenum (E), glandular stomach (F), and bursa fabricius (G) of experimentally infected ducks (ns, *P > *0.05; *, *P < *0.05; **, *P < *0.01; ***, *P < *0.001; ****, *P < *0.0001; *t* test).

We determined viral replication in the tissue organs of infected ducks by qPCR at various time points postinfection and found that replication of ΔLORF3 was consistently reduced compared to the WT virus (Fig. 9). The spleen of ΔLORF3-infected ducks had the highest viral content at the early stage of infection, and the average viral copies in the organs were approximately 44-fold and 23-fold lower than those of ΔLORF3 Rev and WT-infected ducks, respectively (Fig. 9C). Viruses multiply in ducks during the onset period. The viral load in ΔLORF3-infected ducks ranged from ~10^6.94^ to 10^9.97^ copies/0.1 g, compared with ~10^8.35^ to 10^11.13^ copies/0.1 g in the WT group and ~10^7.88^ to 10^11.08^ copies/0.1 g in ΔLORF3 Rev group (Fig. 9A to G). Ducks were resistant to death in the late stage of virus infection, and the number of viruses in the ducks was significantly reduced (Fig. 9A to G). Overall, the replication level of DPV in ducks was reduced after the knockout of LORF3 protein compared with WT, and the reduced viral replication ability was one of the reasons why ducks did not get sick and die. In conclusion, the virulence of the virus was significantly reduced after the *LORF3* gene knockout, and the *LORF3* gene is a DPV virulence gene.

## DISCUSSION

Like other herpesviruses, DPV has a large genome, most of which share homology with other alphaherpesviruses and play roles in various processes of the viral life cycle. However, the research on the pathogenic mechanism of DPV is not deep enough, and the functions of many proteins have not been verified in DPV, as DPV whole-genome sequencing studies have identified some potential genes, such as *LORF3*, which are also unique to the avian herpesvirus based on sequence alignments (21, 22). Whether the putative specific gene is translated into protein and its role in the viral life cycle remain unknown. Using the virulent DPV strain CHv, we concluded that the LORF3 protein is produced during viral infection, which was the first time it was characterized. Herpesvirus genes can be divided into three categories according to their transcription sequence, immediate early genes (IE), early genes (E), and late genes (L), which are sequentially expressed in a cascaded manner (23, 24). Our results indicated that *LORF3* may be an early gene that encodes viral structural proteins and is predominantly located in the nucleus in DPV-infected DEFs. Currently reported DPV structural proteins are encoded by late genes, including *UL41*, *UL47*, *US5*, and *US10* (25–28), which promote the replication of the virus by inhibiting the production of duck IFN-β or inhibiting cell apoptosis. Although the vast majority of structural proteins reported so far are encoded by late genes, very few early genes encode structural proteins, such as US3, UL13, and UL23, which play important roles in herpesvirus pathogenesis (29, 30). The DPV US3 protein plays a role in the cell-to-cell transmission of viruses and virion nuclear export. Likewise, DPV lacking UL13 has attenuated *in vitro* replication (30, 31). For HSV, viral kinases US3 and UL13 promote the expression of late viral genes by recruiting host factors to the DNA replication site in the nucleus. In addition, UL13 and US3 protein kinases interact to promote the assembly and release of mature, infectious virions. Thence, the LORF3 protein may promote viral replication, both in terms of the properties of the early protein and the function of the structural protein.

Next, we investigated this novel uncharacterized *LORF3* gene in the context of viral replication. We generated a *LORF3* knockout mutant virus. The mutant virus lacked pLORF3 expression, could replicate efficiently in infected DEFs, and elicited cytopathic effect (CPE) similar to WT. Using the ΔLORF3 mutant virus, we could demonstrate that pLORF3 is dispensable for viral replication *in vitro* and *in vivo*. The viral replication cycle includes adsorption, invasion, viral nucleic acid replication, assembly of nucleocapsids, maturation, virus release, and cell-to-cell spread (32). In the early stage of virus infection, the envelope glycoproteins are adsorbed to specific receptors on the cell surface, the virus envelope fuses with the cell membrane, and the virus enters the cell and begins uncoating. Then, the virus’s genetic information is conveyed to the nucleus to synthesize various components and enzymes required for virus assembly. Finally, the newly synthesized viral components are gradually matured and packaged into complete viral particles in infected cells and are excreted outside the cell through exocytosis or the endoplasmic reticulum system (33). This experiment judged that knocking out the LORF3 protein would inhibit the late stage of virus replication. However, further verification is needed to determine the function of the LORF3 protein in the viral life cycle. Likewise, the DPV genes, including *US3*, *UL41*, *UL11*, *gI*, *gJ*, and *LORF5*, have also been described to positively regulate the later stages of viral replication, such as spread (34–36). Furthermore, it is worth mentioning that membrane fusion is key to the movement of viruses between cells, and glycoproteins on the surface of viral envelopes can fuse cell membranes to form stomata that allow the transfer of viral genomes between cells (37). Although it is not clear how LORF3 regulates viral replication, there is no doubt that replication rate is an important factor affecting viral virulence.

Furthermore, we set out to investigate whether this novel specific protein could play a role in DPV pathogenesis. DPV, after depletion of LORF3 protein expression, did not cause disease and atrophy of the thymus in susceptible animals. LORF3 is closely related to the pathogenicity of DPV, and the reduced replication level of deletion mutants is one of the reasons why the disease does not occur. This situation also occurs in the DPV *gE* gene. Whether the intracellular or extracellular domains of *gE* are knocked out, the clinical pathogenicity and lethality of recombinant strains in ducks are greatly reduced (38). Among them, the intracellular domain of *gE* facilitates pUL11 incorporation into viral particles. In our research, elimination of the LORF3 protein effectively suppressed DPV infection-induced systemic sepsis, apart from the spleen. As we all know, the spleen is the largest peripheral lymphoid organ and an important site of immune responses. The blood-spleen barrier (BSB) plays a significant role in resisting various pathogens (39, 40). This fierce protective response may have resulted in the “glorious injury” of the spleen organ. Commercial MDV vaccines can cause lymphatic organ atrophy in chickens after immunization, and MDV-attenuated vaccines that do not cause lymphatic organ atrophy in chickens are under continuous research and development (13), suggesting that ensuring that the host animal’s lymphoid organs are not damaged may be one of the necessary conditions for a safe vaccine. In our study, *LORF3* ablation effectively inhibited DPV-induced pathological atrophy of the thymus. *LORF3* has the potential to be a target gene for genetic engineering vaccines.

In the process of intracellular replication, the virus produces viral proteins and their related intermediates, which interfere with the cell survival state or cell function state and damage the cell’s enzyme biochemical system and the activity of cell biomolecules, resulting in loss of cell function or death (41). Studies have reported that a large number of virulence-related viral proteins are involved in the regulation of the host cell microenvironment. For instance, RLORF4 could regulate NF-κB pathway activation (18, 19), and RLORF7 could interact with other cellular proteins, including HDAC1/2 and the multifunctional protein and “guardian of the genome” p53, and induce host cell apoptosis (20, 42). In addition, transcriptional unit of the major viral oncogene encoding the Meq protein and the latency-associated transcripts (LATs) also regulate viral virulence (43). Our study proves that LORF3 does contribute to the pathogenesis and pathogenicity of DPV, but we still do not know how LORF3 functions. Further exploring the regulatory mechanism of the *LORF3* gene will help us better understand it and find its target.

In conclusion, we have identified a novel and unique avian herpesvirus protein, LORF3, that is not essential for viral replication *in vivo* and *in vitro*. Furthermore, we demonstrate that the deletion of pLORF3 significantly reduces disease morbidity and mortality. In conclusion, our data provide a basis for further evaluation of the pathogenic mechanism of this novel LORF3 protein and the development of DPV vaccines.

## MATERIALS AND METHODS

### Cells and viruses.

DEFs were maintained in minimal essential medium (MEM; Gibco) supplemented with 10% newborn bovine serum (NBS), 100 U/mL penicillin, and streptomycin and incubated at 37°C in a 5% CO_2_ humidified incubator. Wild-type DPV CHv (5) was generated in our laboratory (GenBank accession no. JQ647509). The DPV CHv-ΔLORF3 deletion strain (here referred to as ΔLORF3) and DPV CHv-ΔLORF3 Rev restorer strain (here referred to as ΔLORF3 Rev) were constructed in our experiments based on the artificial chromosome rescue platform of duck plague recombinant virus constructed in our laboratory.

### Antibodies.

The rabbit polyclonal antibodies of UL29, UL47, VP5, UL28, ICP27, and LORF3 were generated by our laboratory. The following antibodies were purchased commercially: anti-Flag mouse monoclonal antibody (MBL, Japan) with a dilution of 1:5,000, horseradish peroxidase (HRP)-conjugated goat anti-mouse IgG, goat anti-rabbit IgG (Bio-Rad) with a dilution of 1:3,000, Alexa Fluor 594 goat anti-rabbit IgG (Thermo Fisher Scientific, USA) with a dilution of 1:1,000, and Alexa Fluor 488 goat anti-mouse IgG (Thermo Fisher Scientific, USA) with a dilution of 1:1,000.

### Plasmid construction.

pET32a(+) and pCAGGS were provided by the Sichuan Agricultural University Avian Diseases Research Center. The expression plasmids pET32a(+)-LORF3-His and pCAGGS-LORF3-3xFlag were used in this study. The DPV LORF3 open reading frame was amplified by PCR and inserted into pCAGGS and pET32a(+) vectors in the frame to produce Flag-LORF3 and His-LORF3. The primer sequences are provided in Table 3.

**TABLE 3:** Sequences and characteristics of PCR and qPCR primers

Primer	Primer sequence (5′–3′)	Gene
Pro LORF3-F	Ccggaattcgccaccatgcgtgtagtgatgatg	pET32a(+)-LORF3-His
Pro LORF3-R	ccgctcgagttaatggtgatggtgatgatgttgtcgcctaatgcggtt	pET32a(+)-LORF3-His
Eu LORF3-F	Ccggaattcgccaccatgcgtgtagtgatgatg	pCAGGS-LORF3-3×Flag
Eu LORF3-R	ggaagatctttacttatcgtcgtcatccttgtaatccttatcgtcgtcatccttgtaatccttatcgtcgtcatccttgtaatcttgtcgcctaatgcggtt	pCAGGS-LORF3-3×Flag
ΔLORF3-Kan-F	Ggacggcctagccaagaagccagacgaactacgcacagcttagggataacagggtaatcgattt	LORF3-deleted targeting fragment
ΔLORF3-Kan-R	Taaccccctcttttatgtcctaggtcgcctgttacccataagctgtgcgtagttcgtctggcttcttggctaggccgtccgccagtgttacaaccaat	LORF3-deleted targeting fragment
LORF3 Rev-F	Atcaagggccttcggacagaggacggcctagccaagaagccagacgaactacgcacagctatgcgtgtagtgatgatgca	LORF3-returned targeting fragment
LORF3 Rev-R	Tgtcctaggtcgcctgttacccatattattgtcgcctaatgcggt	LORF3-returned targeting fragment
LORF3 Rev-Kan-F	Tatgggtaacaggcgacctaggacataaaagagggggttagtaatgttgatagg gataacagggtaatcgat	LORF3-returned targeting fragment
LORF3 Rev-Kan-R	Gcacctacaatttacacctcggcgacaacatcaacattactaaccccctcttttatgtcctaggtcgcctgttacccatagccagtgttacaaccaat	LORF3-returned targeting fragment
ΔLORF3-F	Ctcttgacaaacaggtct	LORF3 deletion identified
ΔLORF3-R	Tcctacaaattacccgacacg	LORF3 deletion identified
UL23-ΔMiniF-F	Ttattaatctcaggagcctgtgtagcgtttataggaagtagtgttctgtcatgatgcctgcaagcggtaacgaaaacgattgttacaaccaattaacc	Deleted MiniF components
UL23-ΔMiniF-R	Ccgctccacttcaacgtaacaccgcacgaagatttctattgttcctgaaggcatattcaacggacatattaaaaattga	Deleted MiniF components
UL30-F	Ttttcctcctcctcgctgagt	*UL30*
UL30-R	Ggccgggtttgcagaagt	*UL30*
DPV probe	Cgcttgtacccaggg	*UL30*
LORF2-F	Tccagagatcgcgggatagt	*LORF2*
LORF2-R	Tgcctcggttgttccaaagt	*LORF2*
LORF3-F	Ggagaagacgctcaccagtt	*LORF3*
LORF3-R	Gcctaatgcggttcggtat	*LORF3*
UL1-F	Gattggcgctcgtgatccta	*UL1*
UL1-R	Gttgaagtctccgaggtccc	*UL1*
ICP4-F	Cgttcgctcagctataccct	*ICP4*
ICP4-R	Ggtccgcttatactgagtcca	*ICP4*
UL29-F	Aacctgcgttcgtctccaat	*UL29*
UL29-R	Gtctctctagtcgcatccgc	*UL29*
UL47-F	Aacggagttgcttggagaaca	*UL47*
UL47-R	Tgggcgatgaaacagagtagg	*UL47*
β-actin-F	Gacatccgcaaagacctg	β-actin
β-actin-R	Aggccaggatggagccgcc	β-actin

### Construction of recombinant viruses.

DPV CHv-ΔLORF3 and DPV CHv-ΔLORF3 Rev mutant viruses utilized in this study were constructed via the bacterial artificial chromosome recombinant duck plague virus gene-editing platform. ΔLORF3-Kan-F/R, LORF3 Rev-F/R, and LORF3 Rev-Kan-F/R were used to amplify ΔLORF3-Kan fragment and LORF3 Rev-Kan fragment by PCR to construct DPV CHv-BAC-ΔLORF3 and DPV CHv-BAC-ΔLORF3 Rev, respectively. The base fragment (I-SceI-BAC-Kan-UL23 fragment) containing the I-SceI restriction site, a homologous arm of the BAC element ori2 gene, and Kan gene and UL23 gene sequence were amplified with UL23-ΔMini-F/R primers, subsequently. DPV CHv-ΔLORF3 and DPV CHv-ΔLORF3 Rev mutants with deletion of BAC-MiniF were constructed by the same method. ΔLORF3-F/R primers were used for PCR amplification, restriction endonucleases EcoRI and KpnI were used for restriction fragment length polymorphism (RFLP) identification, and Western blotting (WB) and immunofluorescence assay (IFA) were used to identify LORF3 protein knockout or reversal.

### RNA extraction and reverse transcription-PCR analysis.

Total RNA was isolated from DPV CHv-infected DEFs at different time points (2, 4, 6, 8, 12, 18, 24, 36, and 48 hpi), and reverse transcription was performed. An uninfected control was included. The quantitative primers ICP4-F/R, UL29-F/R, UL47-F/R, LORF2-F/R, LORF3-F/R, UL1-F/R, and β-actin-F/R were designed with Oligo 7 (Table 1). Quantitative reverse transcription-PCR was performed in a 10-μL reaction volume containing 5 μL of SYBR green mix (TaKaRa, Japan), 0.5 μL of each primer, 1 μL of cDNA, and 3 μL of RNase-free water, and all test data were set for 3 repetitions. Triplicate experiments were performed to analyze *LORF2*, *LORF3*, and *UL1* gene expression, and the relative transcript levels were calculated using the threshold cycle (2^−ΔΔ^*^CT^*) method. We used β-actin as the internal reference gene, *ICP4* as the IE gene control, *UL29* as the E gene control, and *UL47* as the L gene control (27, 44–46).

### Virion purification.

The CHv strain was inoculated into 9-day-old duck embryos. After the embryo body died, allantoic fluid was aspirated and centrifuged at 12,000 rpm for 20 min, and the supernatant was taken for later use. We added 1 mL of 30% sucrose solution into the centrifuge tube, and the supernatant of allantoic fluid was gently added into the centrifuge tube and centrifuged at 30,000 rpm for 2 h. The DPV particles were precipitated with phosphate-buffered saline (PBS), and the purified DPV particles were sent to Sangon Biotech Company (Shanghai, China) for mass spectrometry analysis.

### Mass spectrometry.

Purified virion samples were boiled for 10 min with 5 × SDS loading sample buffer and then analyzed by 10% SDS-PAGE. The whole gel was stained with Coomassie brilliant blue (Sigma) and then sent to Sangon Biotech Company for liquid chromatography-tandem mass spectrometry (LC-MS/MS) analysis.

### Western blotting.

For Western blotting (WB), lysates were separated by SDS-PAGE, and then the proteins were transferred to a polyvinylidene difluoride (PVDF) membrane (Millipore, MA, USA), which was subsequently blocked with a blocking buffer (5% skim milk and 0.1% Tween 20 in PBS) for 2 h at room temperature. The membrane was incubated overnight at 4°C with rabbit anti-LORF3, anti-UL29, anti-UL47 or rabbit anti-VP5, and rabbit anti-UL28 monoclonal antibodies at 1:1,000 and mouse anti-Flag polyclonal antibody at a dilution of 1:3,000. Then, the membrane was washed three times with PBS with Tween 20 (PBST) and incubated with IPkine HRP AffiniPure goat anti-rabbit IgG light chain at a dilution of 1:3,000 and HRP-conjugated goat anti-mouse IgG (1:3,000) secondary antibodies for 1 h at 37°C. The membrane was washed three times with PBST, and the signals were developed using an enhanced chemiluminescence (ECL) kit (TaKaRa, Japan).

### Pharmacological inhibition reaction.

The procedure was carried out as previously described (43, 47). Nucleic acid synthesis inhibitor GCV and protein synthesis inhibitor CHX were used to determine the *LORF3* gene type. Specifically, DEF monolayer cells were treated with 250 μg/mL CHX and GCV and simultaneously infected with DPV at a multiplicity of infection (MOI) of 0.01 and cultured at 37°C for 18 h, and total RNA was isolated and reverse transcribed into cDNA using a reverse transcription kit. cDNA was used for PCR analysis, and the product was identified by agarose gel electrophoresis.

### Immunofluorescence analysis.

The monolayer DEF cells grown on the slide were washed 3 times with PBS and fixed overnight with 4% prechilled paraformaldehyde in PBS after transfection or infection. For indirect IFA, the fixed cells were permeabilized with 1% Triton X-100 in PBS for 20 min at room temperature and mixed with 300 μL of blocking buffer (5% bovine serum albumin in PBST) in a humidified chamber. They were then incubated together at 37°C for 2 h. Then, the cells were combined with primary antibodies (mouse anti-Flag, rabbit anti-hemagglutinin [HA], and rabbit anti-LORF3, diluted to 1:3,000, 1:3,000, and 1:800, respectively) and Alexa Fluor-conjugated secondary antibodies (diluted at 1:1,000) in 1% PBST-bovine serum albumin (BSA) buffer at 37°C for 60 min. We examined the sample under a Nikon H550 L fluorescence microscope.

### Multistep growth kinetics *in vitro*.

Replication properties of recombinant viruses were assessed by multistep growth kinetics as described previously (15, 31). Briefly, DEF cells in 24-well plates were infected with an MOI of 0.01 of indicated viruses; samples were collected at 12, 24, 48, 72, and 96 h postinfection (hpi) and stored at −80°C. Virus titers in samples were determined to assess the level of virus growth by determining the 50% tissue culture infectious dose (TCID_50_). Statistical analyses were performed using GraphPad Prism version 8 (San Diego, CA, United States), and data were considered significantly different if the *P* value was ≤0.05. Growth kinetics data were repeated three independent times. Asterisks indicate significant differences compared to WT virus (***, *P < *0.001; **, *P < *0.01; *, *P < *0.05).

### Real-time fluorescence quantification PCR.

Viral DNA was extracted from samples using the RTP DNA/RNA virus minikit (Stratec, Berlin, Germany) and measured by qPCR. Primers and probes specific for the DPV UL30 designed previously in our laboratory are shown in Table 3. Ex Taq premix (probe qPCR) (TaKaRa, Dalian, China) was used to determine viral DNA copies. qPCR amplifications were performed under the following conditions: 95°C for 30 s, followed by 40 cycles at 95°C for 5 s and 60°C for 30 s. Then, the qPCR products were quantified by comparison with the established standard curve of the laboratory. All reactions were performed in triplicate and at least three independent experiments.

### Viral adsorption, penetration, replication, and release assays.

**(i) Adsorption.** DEFs were cultured in 6-well plates, and the cells were precooled at 4°C for 2 h after they were well grown. Then, the cells were washed with 4°C precooled PBS and infected with virus at an MOI of 0.1. After infection, the cells were placed at 4°C with slight shaking for 2 h and finally washed 5 times with precooled PBS, and cell samples were collected for virus copy number determination.

**(ii) Penetration.** We infected the virus according to the above-described adsorption operation; after 2 h, the supernatant was discarded and replaced with maintenance solution, and the cells were placed in a 37°C cell incubator for 1 h. Finally, the cells were washed 5 times with precooled PBS, and cell samples were collected for virus copy number determination.

**(iii) Replication.** DEFs were infected with virus at an MOI of 1 or 0.01 for 6 h and replaced with cell maintenance medium to continue the culture. Cells were collected at another 7, 8, 9, and 10 hpi for virus copy number determination.

**(iv) Release.** DEFs were infected with viruses at an MOI of 1 for 16 h and replaced with maintenance medium. The supernatant was collected 1 h, 2 h, 3 h, and 4 h after the maintenance solution was replaced to detect the virus TCID_50._

### The cytopathic plaques by crystal violet staining.

Cell-to-cell spread of the mutant viruses was measured by plaque size assays (48). DEF cells in 6-well plates were infected with indicated viruses at an MOI of 0.001. After incubation for 2 h at 37°C, 1.5% methylcellulose (Solarbio, Beijing, China) was added to cover the cells. The cytopathic plaques by crystal violet staining when the cells were infected for 5 days. In particular, the medium was discarded, cells were fixed with 500 μL of precooled 4% paraformaldehyde for 20 min and washed twice with sterile PBS, 500 μL 0.5% crystal violet was added for staining for 30 min, and the cells were then rinsed with tap water to remove the staining solution and observe and count the plaques. The average plaque size was measured using Image-Pro Plus software (Bio-Rad, CA, USA). The plaque sizes of the deletion virus and the reverted virus were calculated and compared with the plaque size of the parental virus set at 100%. Data were considered significantly different if the *P* value was ≤0.05 (***, *P < *0.001). All reactions were performed in triplicate and with at least three independent experiments.

### *In vivo* experiment.

To investigate the role of LORF3 in DPV replication and pathogenesis *in vivo*, 14-day-old ducklings were intramuscularly injected with 10^4^, 10^5^, and 10^6^ TCID_50_ of the parental wild-type CHv (WT; *n* = 10), ΔLORF3 Rev (*n* = 10), and ΔLORF3 (*n* = 10), respectively. Ducklings were monitored daily for clinical signs of disease that include severe ataxia, paralysis, torticollis, and somnolence. Furthermore, the anal temperature and body weight of ducks and mortality were also monitored daily. During and after termination of the experiment, ducklings that died of disease or were humanely euthanized were examined for histopathological.

### Quantification of DPV genome copies in ducking viscera.

Heart, liver, spleen, thymus, duodenum, glandular stomach, and bursa of Fabricius samples were taken from the infected animals at 2, 6, and 10 dpi of each group to assess virus replication *in vivo*. DNA was isolated from all samples using the blood, cell, and tissue genomic DNA extraction kit (Qiagen; catalog no. DP304) according to the manufacturer’s instructions. DPV genome copies were measured by qPCR as described above.

### Statistical analysis.

Statistical analyses were performed using GraphPad Prism version 8 (San Diego, CA, USA), and data were considered significantly different if *P* values were <0.05. Growth kinetics and plaque size data were analyzed using one-way analysis of variance (ANOVA).

### Ethics statement.

All animal experiments were conducted in accordance with approved guidelines. One-day-old Peking ducks were purchased from a farm operated by Sichuan Agricultural University (Sichuan, China). All experimental ducks did not contain DPV and were negative for DPV antibodies. All of the ducks were housed in the animal facility at Sichuan Agricultural University, Chengdu, China. The study was approved by the Committee of Experiment Operational Guidelines and Animal Welfare of Sichuan Agricultural University (approved permit number XF2014-18).

### Data availability.

The sequence of DPV CHv was deposited into GenBank under accession number JQ647509.
